# Efficacy and safety of hepatic arterial infusion chemotherapy combined with tyrosine kinase inhibitors and immune checkpoint inhibitors in the treatment of advanced hepatocellular carcinoma with portal vein tumor thrombosis in the main trunk

**DOI:** 10.3389/fonc.2024.1374149

**Published:** 2024-07-15

**Authors:** Qi Liu, Ying Zhang, Jingwen Zhang, Luhao Chen, Yi Yang, Yan Liu

**Affiliations:** Department of Interventional Radiology, Harbin Medical University Cancer Hospital, Harbin, China

**Keywords:** hepatocellular carcinoma, portal vein tumor thrombus, HAIC, TKI, ICI

## Abstract

**Purpose:**

To evaluate the efficacy and safety of mFOLFOX-based hepatic arterial infusion chemotherapy (HAIC) combined with tyrosine kinase inhibitors (TKIs) and immune checkpoint inhibitors (ICIs) in the treatment of advanced hepatocellular carcinoma (HCC) with portal vein tumor thrombosis (PVTT)

**Methods:**

This retrospective study included patients who received mFOLFOX-based HAIC combined with TKIs and ICIs from January 2021 to January 2023. The primary outcome was the objective response rate of PVTT response, and the secondary outcomes were 6-month, 1-year survival rate, overall survival (OS), and corresponding adverse events and complications were also evaluated. PVTT responses were assessed using ITK-SNAP software.

**Results:**

A total of 37 patients were included in the analysis, 18.92% achieved a complete response and 56.76% achieved a partial response in PVTT response. The objective response rate (ORR) of PVTT was 75.68%. The 6-month survival rate was 89%, the 1-year survival rate was 66%, and the median OS was 15.8 months. In univariate analysis, Child-Pugh score (P=0.010) was important factor for predicting OS; in multivariate analysis, Child-Pugh score (P=0.015, HR= 3.089, 95%CI: 1.250–7.633) was the important factor for predicting OS. In terms of adverse reactions, the most common adverse reactions associated with HAIC are pain and thrombocytopenia associated with oxaliplatin.

**Conclusion:**

FOLFOX-based HAIC combined with TKIs and ICIs induced an objective response rate of 75.68% in PVTT.

**Clinical signicance:**

FOLFOX-based HAIC combined with TKIs and ICIs provides more treatment options for PVTT.

## Highlights

- The treatment effect of PVTT was accurately evaluated using ITK-SNAP software.
- Verified the safety and effectiveness of HAIC combined with ICIs and TKIs in the treatment of PVTT.
- Providing more treatment options for intrahepatic tumors.

## Introduction

Hepatocellular carcinoma (HCC) comprises for 75–85% of primary liver cancer cases and is the fourth leading cause of annual cancer deaths worldwide (1). Portal vein tumor thrombosis (PVTT) is the most common form of Macrovascular invasion (MVI) of advanced HCC, which occurs in In 10–60% of patients with HCC (2). If only treated with supportive care, the median survival of HCC with PVTT is about 2.7 months (3, 4). PVTT is related to poor prognosis probably because of the intensified risk of tumor spread, increased portal pressure inducing variceal bleeding, and reduced portal flow and subsequent jaundice, ascites, hepatic encephalopathy, and hepatic failure (5). Especially when the PVTT invades the main portal vein (VP3, VP4), the prognosis is very poor. Many experiments exclude patients with VP3 and VP4. Any HCC patient with PVTT is classified as advanced (Barcelona Clinic Liver Cancer stage C) and is suitable for palliative systemic therapy (6–8). At this stage, atezolizumab plus bevacizumab, sorafenib, and lenvatinib are considered recommended first-line treatment. Recently, the combination of the immune checkpoint inhibitors (ICIs) atezolizumab plus theanti-vascular endothelial growth factor (VEGF) antibody bevacizumab as the first-line treatment of advanced HCC showed better overall survival (OS) compared with sorafenib, and this systemic combination therapy has been recommended as the first-line therapy for HCC patients with PVTT (9). Immunotherapy has demonstrated safety and efficacy in later lines of therapy as well, and ongoing trials are investigating novel combinations of ICIs and tyrosine kinase inhibitors (TKIs) (10).

Owing to the physiologic basis of hepatic arterial bloodsupply to the PVTT, HAIC is expected to increase the local drug concentration, thereby increasing the therapeutic efficacy of PVTT (11). The study of HAIC plus mFOLFOX (oxaliplatin, leucovorin, 5-F uracil) regimen in the treatment of unresectable HCC showed that It has shown good results, especially in patients with BCLC stage C, with a median OS of 14.5 months (12–14).

Based on these studies, we speculate that HAIC (mFOLFOX) combined with TKIs and ICIs may bring greater therapeutic benefits to patients with advanced HCC with VP3 or VP4. Therefore, the purpose of this study we conducted was to evaluate the efficacy and safety of HAIC combined with TKIs and ICIs in the treatment of PVTT (vp3, vp4).

## Methods

### Study design and patient selection

**Figure 1:**
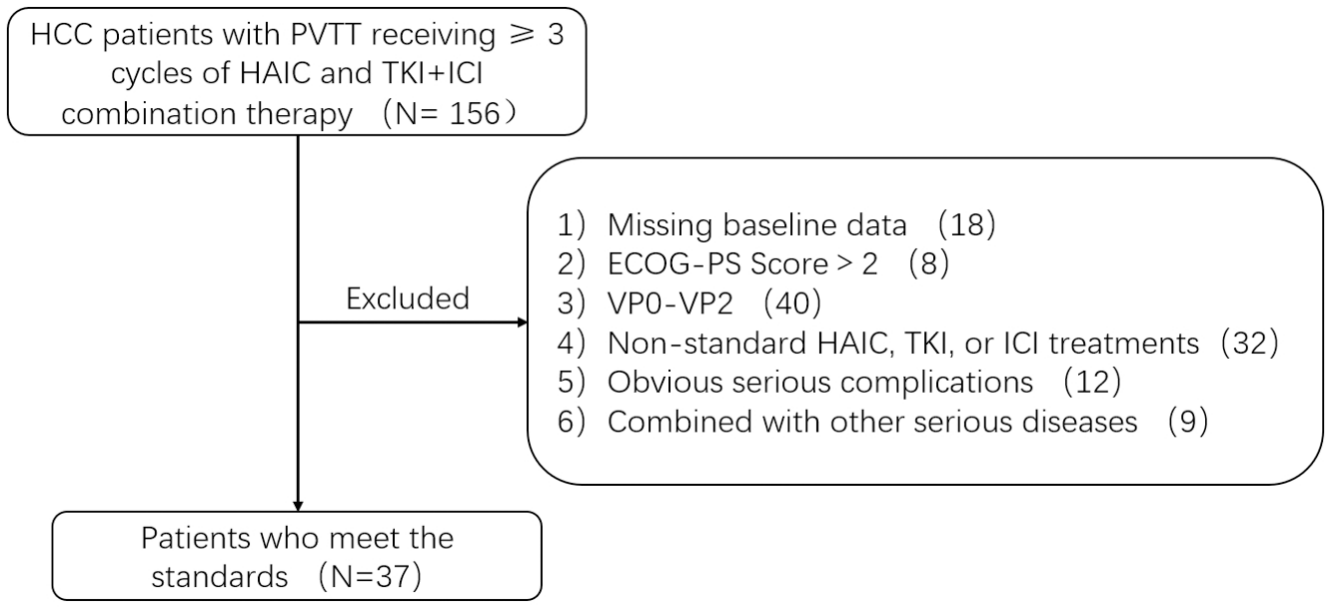
The research flow chart.

From January 2021 to January 2023, we retrospectively analyzed 156 patients who received ≥3 cycles of HAIC and TKIs+ICIs combined therapy in our hospital. This retrospective study was approved by the local ethics committee and the written informed consent form was waived. The research flow chart is shown in **Figure 1**.

Inclusion criteria: 1) Age ≥ 18 years old; 2) Patients with HCC diagnosed by pathology or imaging; 3) Patients with VP3 or VP4 complications diagnosed by enhanced computed tomography (CT) or enhanced magnetic resonance imaging (MRI); 4) patients with tolerable liver function (Child−Pugh score 5−7) at admission; 5) Patients receiving long-term combined treatment of TKIs and ICIs; 6) Patients receiving more than three cycles of HAIC treatment.

Exclusion criteria: 1) Patients with incomplete baseline data; 2) patients with Eastern Cooperative Oncology Group−performance status (ECOG−PS) >2; 3) Patients with VP0-VP2 in the Japanese VP classification; 4) Patients who did not receive TKIs and ICIs treatment at the same time; 5) Patients with obvious complications; 6) Patients with other serious diseases.

Treatment programs:

### HAIC process

**Figure 2:**
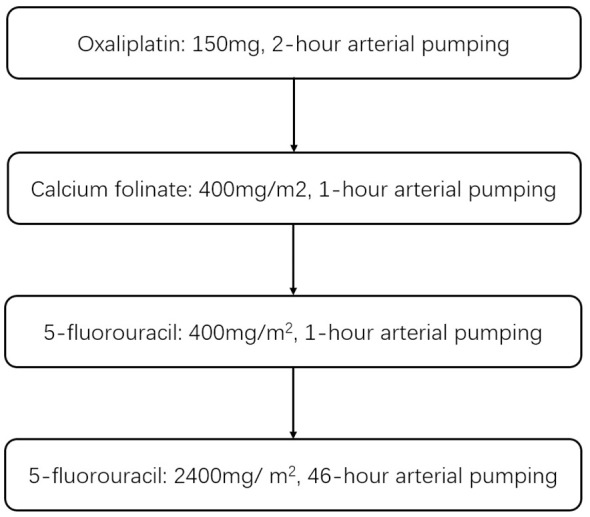
Improved FOLFOX scheme.

After routine preoperative preparations, the Seldinger technique was used to insert the 5F catheter sheath into the femoral artery. Tumor feeding branches were identified by hepatic arteriography. The tip of catheter was placed at the proper heptic artery, the gastroduodenal artery and right gastric artery should be embolized if necessary. The catheter was fixed, then the drugs (oxaliplatin, leucovorin, and fluorouracil) were pumped in. The detailed method is shown in **Figure 2**. Treatment was repeated every 3–4 weeks and continued until untreatable progression or intolerable treatment-related toxicity.

### TKI+ICI drugs

The patients started to use TKIs (Lenvatinib, donafinib, or regorafenib)after HAIC treatment. The measurement and frequency are judged strictly according to the patient’s own condition and drug instructions. ICIs (camrelizumab or sintilimab) 200 mg was initially administered intravenously in 1 week after HAIC of a 21-day cycle based on the condition of the patient.

### Follow-up and repeat treatment

Each follow-up visit included a detailed history, physical examination laboratory tests, enhanced abdominal CT (three-phase), liver-enhanced MRI, and Chest enhanced CT. All patients were evaluated after 2–3 cycles of HAIC. All patients without disease progression continued to receive HAIC.

### Evaluation

The primary outcome of this study is the PVTT remission rate. other evaluation indicators were also analyzed, such as OS, 6-month survival rate, and 1-year survival rate. We collected dynamic contrast-enhanced CT or contrast-enhanced MR Images of the patients before and after treatment. The PVTT images were delineated by using ITK-SNAP 5.2.1 software (15, 16) [open source software, ITK-SNAP Home (www.itksnap.org)], and the volume of PVTT was accurately calculated. The PVTT response was defined based on the volume change in the PVTT before and after treatment. Two imaging experts independently reviewed the enhanced CT or MRI images obtained at baseline and post treatment follow-up. Complete response (CR): PVTT disappeared completely; Partial response (PR): PVTT volume decreased by more than 50%; Stable disease (SD): PVTT volume decreased by less than or equal to 50% or increased by no more than 25%; Progressive disease (PD): PVTT volume increased by more than 25%. Adverse events were evaluated according to the CTCAE version 5.0.

### Additional treatment

After good efficacy with PVTT, we offered additional treatment (TACE) for their intrahepatic tumors on top of HAIC for some patients with a PS score of 0–1 and good liver function.

### Statistical analysis

All statistical analyzes were performed using SPSS 26.0 software. Univariate and multivariate analyzes of PVTT responses were performed using logistic regression. Life tables and Kaplan-Meier survival curves were used to estimate 6-month survival, 1-year survival, and overall survival. The predictors of OS were analyzed by univariate and multivariate analysis by COX proportional hazards regression. P<0.05 was considered statistically significant.

## Result

### Patient baseline characteristics

A total of 37 patients were enrolled in this retrospective cohort study. **Table 1** summarizes the data characteristics of the 37 patients before initial treatment. The median age of the patients was 56 years (range 42–73 years), and 34 (92%) patients were male. Child-Pugh scores of 5, 6, and 7 were noted in 23 (62%), 9(24.0%), and 5 (14%) patients, respectively. Eastern Cooperative Oncology Group−performance status (ECOG−PS) scores of 0 and 1 were noted in 12 (32%) and 25 (68%), respectively. Twenty-eight (76%) patients were positive for hepatitis B antigen, 2 (5%) patients were positive for hepatitis C antibody, and 7 (19%) patients had no hepatitis. The median volume of PVTT before treatment was 22160 mm^3^ (range 317.1–147300 mm^3^). HCC with vp3 or vp4 was presented in 23 (62%) and 14 (38%) patients, respectively. The median size of liver tumors was 90mm (range 9–162mm). The median AFP level was 979 ng/mL (range 0.78–451613 ng/mL). Four patients were previously treated with radio frequency (RF) ablation. Five patients were previously treated with TACE. Five patients were previously treated with surgery. Two patients were previously treated with sorafenib. All patients were suffering from cirrhosis.

**Table 1:** Baseline characteristics of patients (N=37).

Features	Median (range) or patients, n
Gender (male/female)	34/3
Age (years)	56 (42–73)
Classification of PVTT (VP3/VP4)	23/14
PVTT volume before treatment (mm^3^)	22160 (317.1–147300)
Tumor diameter (mm)	90 (9–162)
AFP (ng/mL)	979 (0.78–451613)
ALT (U/L)	44 (14–307)
AST (U/L)	64 (19–182)
TBIL (umol/L)	20.9 (6.4–44.6)
ALB (g/L)	37.7 (25.9–44.9)
Plt (10^9^/L)	157 (81–262)
PT (s)	12.5 (10.8–14.2)
Child-Pugh Score (5/6/7)	23/9/5
PS Score (0/1)	12/25
Extrahepatic metastasis (Yes/No)	3/34
Hepatitis (HBV/HCV/No)	28/2/7
TKIs (Donafinib/Lenvatinib/Regorafenib)	13/23/1
ICIs (Camrelizumab/Sintilimab)	26/11
Previous treatment (RF/TACE/Surgery/Sorafeni/No)	4/5/5/2/21

PVTT, Portal vein tumor thrombosis; AFP, Alpha-fetoprotein; ALT, Alanine transaminase; AST, Aspartate transaminase; TBIL, Total bilirubin; ALB, Serum albumin; Plt, Platelet; PT, prothrombin time; PS Score, Eastern Cooperative Oncology Group−performance status; TKIs, Tyrosine kinase inhibitors; ICIs, Immune Checkpoint Inhibitors; RF, Radio frequency.

### PVTT response and factors affecting PVTT response

**Figure 3:**
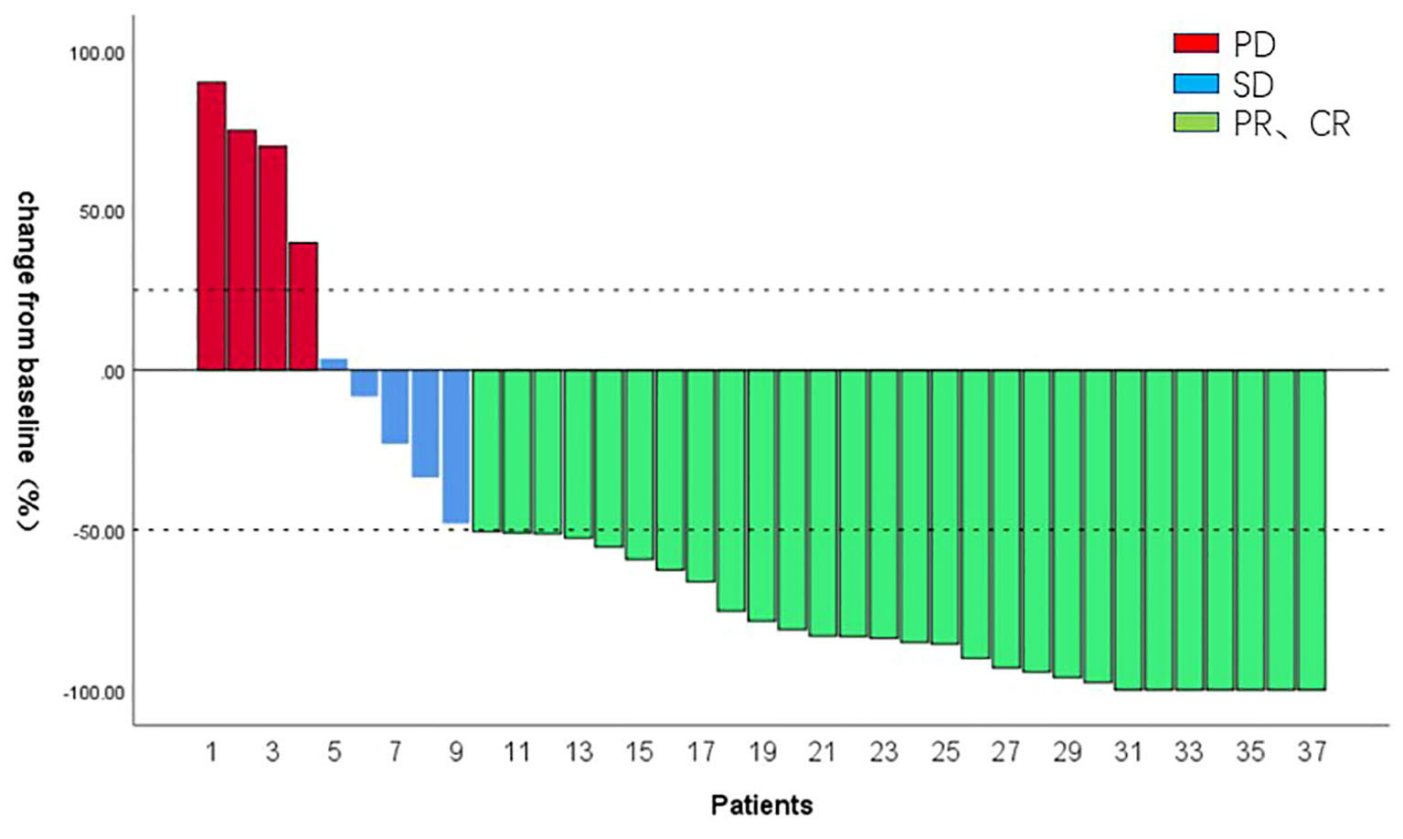
Objective response of HAIC combined with TKI and ICI for PVTT.

**Figure 4:**
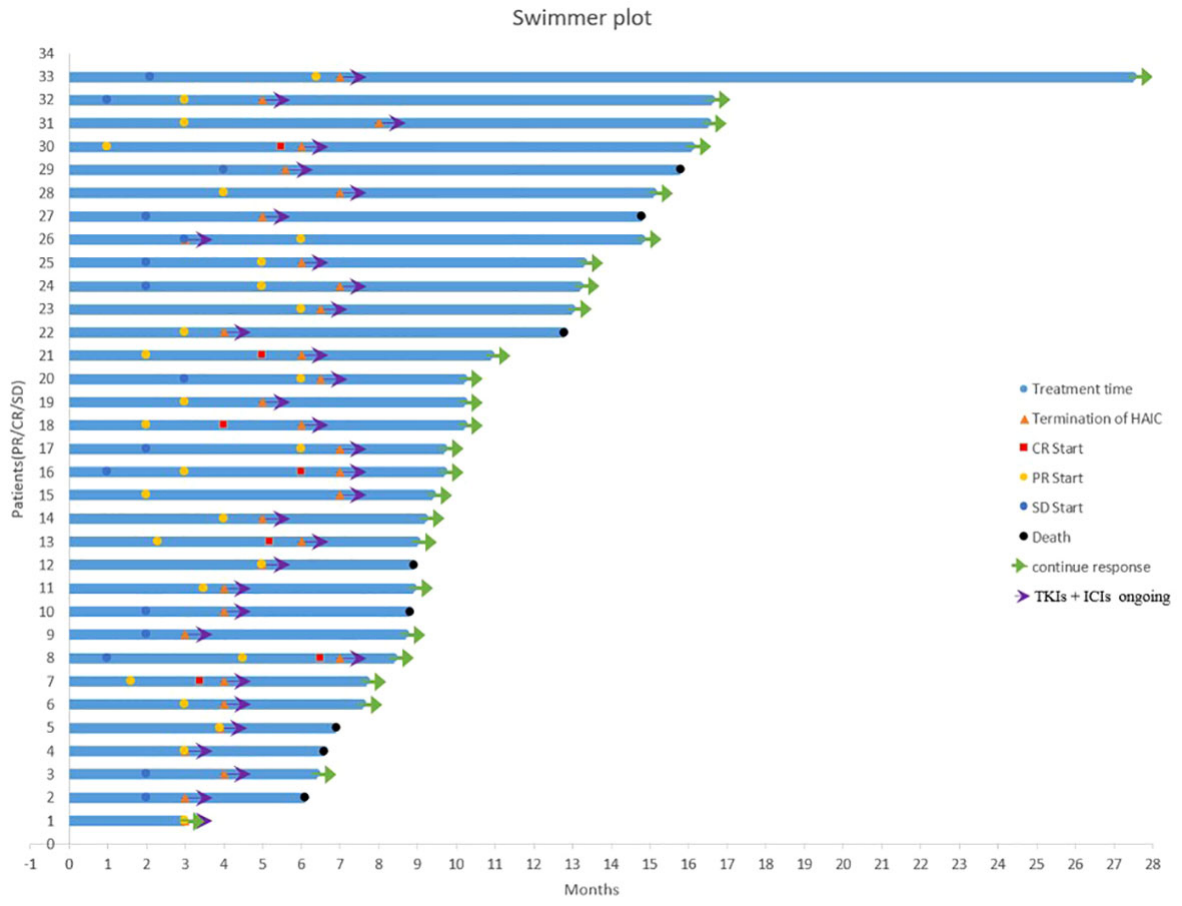
Swimmer plot for treatment duration (Patients of PR/CR/SD).

**Figure 5:**
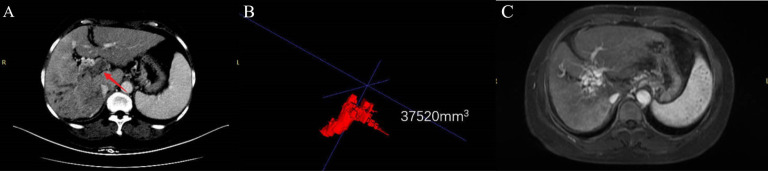
A typical case. A 55-year-old female patient was diagnosed with advanced hepatocellular carcinoma with VP4. Combined with the pre-treatment panel **(A)**, contrast-enhanced CT in the portal venous phase was obtained, showing both the main portal vein and its right branch Filled with PVTT (arrow). Panel **(B)** is the PVTT image delineated by ITKs, and the PVTT volume is obtained to be 37520 mm3. Panel **(C)** is an enhanced MR image after 5 cycles of HAIC (mFOLFOX) combined with donafenib and camrelizumab. It shows that the portal vein tumor thrombus disappeared and portal vein cavernous changes appeared, and the PVTT response reached CR.

All patients received ≥3 cycles of HAIC treatment with a median of 5 cycles (range 3–8 cycles). After combination therapy, the PVTT response of all patients was shown in **Figure 3**. In terms of PVTT response rates, 7 patients (18.92%) achieved complete responses. Among them, four patients had received HAIC combined with lenvatinib+calerizumab and three patients received HAIC combined with donafinib+sinetizumab. Twenty-one patients (56.76%) achieved partial responses. Five patients (13.51%) had stable disease and four patients (10.81%) had progressive disease. The objective response rate (ORR) of PVTT was 75.68%, and the disease control rate (DCR) of PVTT was 89.19%. The follow-up process of the patient with controlled PVTT (SD+PR+CR) was shown in **Figure 4**. A typical case was shown in **Figure 5**.

**Table 2** summarizes the results of the analysis of the relationship between the PVTT response and covariates. Unfortunately, all the covariates were not significantly associated with PVTT response in either univariate or multivariate analyses.

**Table 2:** Univariate and multivariate analyzes for predicting response to PVTT after treatment.

Parameters	Single factor	Multiple factor
	P value	P value
Age (>56/≤56 years)	0.297	0.428
PVTT volume before treatment (>22160/≤22160mm^3^)	0.297	0.093
Tumor diameter (>90/≤90mm)	0.395	0.234
AFP (>979/≤979 ng/mL)	0.635	0.963
Child-Pugh (5/6/7)	0.217	0.331
HBV/HCV (Yes/No)	0.861	0.841
TKIs (Donafinib/Lenvatinib/Regorafenib)	0.389	0.378
ICIs (Camrelizumab/Sintilimab)	0.650	0.262

PVTT, Portal vein tumor thrombosis; AFP, Alpha-fetoprotein; HBV, hepatitis B virus; HCV, hepatitis C virus.

### OS and its prognostic factors

**Figure 6:**
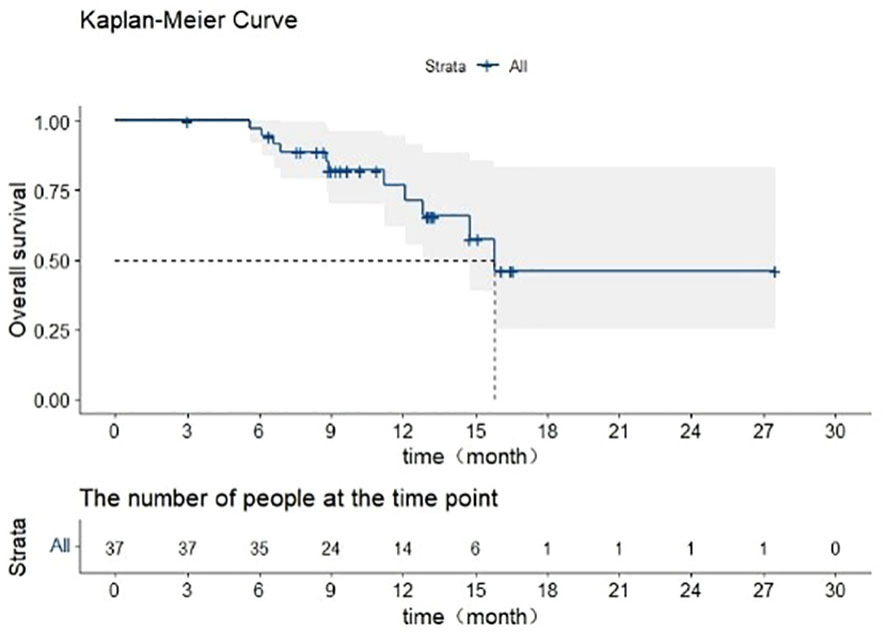
OS of all patients.

The median OS of all patients was 15.8 months (**Figure 6**), the 6-month survival rate was 89%, and the 1-year survival rate was 66%. In univariate analysis, Child-Pugh score (P=0.010) was the important factor for predicting OS; in multivariate analysis, Child-Pugh score (P=0.015, HR= 3.089, 95%CI: 1.250–7.633) was still important independent factor for predicting OS (**Table 3**).

**Table 3:** Univariate and multivariate analyzes for predicting OS.

Parameters	Single	Multiple factor		
	P value	HR	95%CI	P value
Age (>56/≤56 years)	0.628			
PVTT volume before treatment (>22160/≤22160mm^3^)	0.099	1.782	0.428–7.424	0.427
Tumor diameter (>90/≤90mm)	0.057	3.542	0.875–9.333	0.076
AFP (>979/≤979 ng/mL)	0.288			
Child-Pugh (5/6/7)	0.010	3.089	1.250–7.633	0.015
Distant metastasis (Yes/No)	0.286			
HBV/HCV (Yes/No)	0.250			
Classification of PVTT (VP4/VP3)	0.101	0.919	0.236–3.583	0.903
Cavernous transformation of portal vein (Yes/No)	0.325			
TKIs (Donafinib/Lenvatinib/Regorafenib)	0.628			
ICIs (Camrelizumab/Sintilimab)	0.297			

The meaning of the red values: P<0.05. PVTT, Portal vein tumor thrombosis; AFP, Alpha-fetoprotein; HBV, hepatitis B virus; HCV, hepatitis C virus.

### Subsequent therapy after combination therapy

Twenty-three (62%) patients had PVTT volumes remission after 1–2 cycles of HAIC treatment, and their intrahepatic tumors were treated with TACE on top of HAIC after a comprehensive evaluation by clinicians. Patients with stable disease continued to be treated with HAIC in combination with TKIs and ICIs. A total of four patients experienced PVTT progression, two of whom were treated with symptomatic supportive therapy, one of whom abandoned treatment, and one of whom died.

### Treatment-related adverse events

**Table 4** summarizes the adverse events that occurred in this study; the most common adverse event was oxaliplatin-related pain during HAIC (30, 80%), which was relieved by slowing down the pumping rate of oxaliplatin in 20 (54%) patients and by slowing down the pumping rate and pain management in 10 (27%) patients. The CTCAE grade 3 or 4 adverse events observed were an increased alanine aminotransferase/aspartate aminotransferase levels (n = 2, 5%), thrombocytopenia (n = 4, 11%), and leukopenia (n = 2, 5%); As to adverse events related to TKIs, the most common adverse event was hand-foot reaction (16 patients, 43%), of which 6 (11%) had grade 3 adverse events; Four (11%) presented with upper gastrointestinal bleeding and corresponding black stool symptoms, and one patient presented with death due to acute upper gastrointestinal bleeding.

**Table 4:** Adverse events and complications.

Adverse reaction events	Any grade N,%	Grade 3 and above N,%
Pain	30 (80%)	10 (27%)
Fatigue	22 (59%)	0
ALT/AST rise	12 (32%)	2 (5%)
Thrombocytopeni	22 (59%)	4 (11%)
Leukopenia	2 (5%)	2 (5%)
Fever	20 (54%)	0
Hypertension	3 (8%)	3 (8%)
Hand-foot reaction	16 (43%)	6 (16%)
Rash	6 (16%)	4 (11%)
Diarrhea	3 (8%)	0
Oral gingival bleeding	9 (24%)	0
Epistaxis	4 (11%)	0
Upper gastrointestinal bleeding	4 (11%)	2 (2%)
Abdominal pain	2 (5%)	0
Death	1 (3%)	1 (3%)

ALT, Alanine transaminase; AST, Aspartate transaminase.

## Discussion

The prognosis of patients with HCC combined with PVTT (VP3, VP4) is extremely poor and has been a thorny issue for oncologists. Invasion of PVTT into the portal trunk reduces the blood supply to the liver parenchyma, resulting in deterioration of liver function, portal hypertension, and possible complications of upper gastrointestinal bleeding, which is the main cause of death for many patients with advanced liver cancer. There is no uniform treatment protocol and there are significant differences in treatment options between the East and West. In the West, systemic therapy is more often chosen for patients with HCC with PVTT. Atezolizumab combined with bevacizumab is included in the first-line treatment of advanced liver cancer. A subgroup analysis of the Imbrave150 study (17) showed a median OS of 7.6 months for A+T in HCC patients with VP4, superior to sorafenib (median OS of 5.5 months). However, the study did not analyze PVTT responses. A retrospective study by Huang et al (18) analyzed the effect of lenvatinib combined with PD-1 antibody on PVTT,of which the ORR was 54.5%. However, the present study added mFOLFOX-based HAIC in addition to TKIs and ICIs combination therapy obtained an ORR of 75.68% for PVTT, which was significantly better than the above study.

In contrast, in the East, local treatment is more often chosen for HCC patients with portal cancer thrombosis. TACE was previously considered a contraindication for unresectable HCC with PVTT. If liver function is good and there is good collateral circulation, TACE can be used as a treatment option for patients with VP3/4 if intraoperative superselection is done to achieve precise embolization and maximize the protection of liver function. However, for patients with severe PVTT, TACE combined with radiotherapy or TACE combined with TKIs is preferred. Consensus-based guidelines in Japan and Taiwan recommend HAIC as one of the treatment options for VP3 and VP4 PVTT (19, 20, 21). A recent multicenter randomized open clinical trial reported that the combination of sorafenib with HAIC containing 5-fluorouracil, calcium folinic acid, and oxaliplatin (mFOLFOX) achieved a longer median OS than sorafenib alone (13.37 vs. 7.13 months), while the present study added ICIs to mFOLFOX-based HAIC in combination with TKIs therapy achieved a longer median OS (15.8 months). Moreover, this study mainly analyzed the effect of combined treatment on PVTT. In previous reports, local treatment or a combination of several local treatments was mainly used in the treatment of PVTT; Kosaka, Y et al (22) analyzed the efficacy of HAIC in combination with radiation therapy (RT) for HCC with VP4 in a retrospective study, of which the median OS was 12.1 months and the ORR was 51.0%,with a significantly higher median OS in PVTT remission subgroup (PR/CR:19.4 months) than in nonresponding subgroup (SD: 14.6 and PD: 4.2 months); Chen et al (23) used the HabibTMVesOpen intravascular radiofrequency ablation catheter to produce positive clinical results with radiofrequency ablation of portal vein cancer thrombi by the percutaneous puncture; SUN et al (24) reported an ORR of 42.1% for PVTT in the group of radioactive ^125^I particle implantation combined with TACE in a study of advanced hepatocellular carcinoma. ^125^I particles implanted into portal PVTT can kill the thrombus and maintain the blood supply to the liver, while further TACE can be performed. In all of the above studies, the median OS of those with remission of PVTT in some studies was significantly higher than those of non-responders. This study was conducted in patients with VP3 and VP4 and mainly evaluated the ORR of PVTT (75.68%). After the PVTT was relieved, precise TACE was performed to the intrahepatic lesion. We performed TACE with strict superselection of the blood supply vessels to the tumor, there was no significant deposition of iodinated oil in the portal vein. Therefore, we believe that additional TACE treatment will not affect the analysis of the therapeutic effect of FOLFOX-based HAIC combined with TKIs and ICIs on PVTT. But,the efficacy of TACE may be masked when analyzing the association between a good PVTT response and significantly prolonged OS. In the COX proportional hazards regression model, Child-Pugh score was an independent prognostic factor affecting OS, and good hepatic function reserve provided a longer OS. A similar view was shown in a recent meta-analysis (25), where poorer hepatic functional reserve shortened OS. However, after each treatment of HAIC combined with TKIs and ICIs, the patient’s hepatic function was somewhat impairment, we recorded it as an adverse event. Good hepatic functional reserve at baseline implies that the liver has the capacity to deal with injury from combination therapy and a high capacity for recovery. However, in this study, we included patients with Child-Pugh 5/6/7 and excluded patients with significant serious complications such as ascites, esophagogastric variceal bleeding, and jaundice. In response to the hepatic impairment caused by the combination therapy, we treated the patients symptomatically with hepatoprotective drugs. Therefore, we only performed COX regression analysis on Child-Pugh scores at baseline and did not include analysis of liver functional reserve in patients on treatment. Moreover, for the evaluation of PVTT, we used ITK-SNAP to outline the images of PVTT before and after treatment and calculate the volume. This calculation method of thrombus volume has not been reported in the literature, but we think that for the irregular solid tumors such as portal vein thrombus, this calculation method is more accurate than the WHO solid tumor evaluation method and the Response Evaluation Criteria In Solid Tumors (RECIST v1.1), but its rationality needs to be further verified. The degree of remission of PVTT was referred to the WHO solid tumor efficacy assessment criteria. PR was set as tumor shrinkage ≧50%, and PD was set as tumor enlargement ≧25%. The rationality of this criterion also needs to be further verified. Also, we have some shortcomings. As a single-center single-arm retrospective study, the number of patients is insufficient; TKIs and ICIs drugs are not uniform; the follow-up time of the included patients is short, and some patients have not reached the OS outcome. Patients with significant comorbidities that prevented them from completing regular therapy were excluded to better represent the therapeutic effect of regular therapy on cancer emboli, which may have led to bias in the assessment of treatment safety in this study.

At present, there are no uniform criteria for assessing the PVTT response, so it is important to reach a consensus on the PVTT response in order to better research.

## Conclusion

In this retrospective study, FOLFOX based HAIC combined with targeted therapies and immunotherapy induced an objective response rate of 75.68% for PVTT, providing more treatment options for PVTT.

HAIC combined with targeted therapies and immunotherapy can significantly prolong the survival of liver cancer patients with VP3/VP4 PVTT.

The safety of HAIC combined with targeted therapies and immunotherapy is acceptable, and can be relieved through symptomatic treatment.

## Data availability statement

The raw data supporting the conclusions of this article will be made available by the authors, without undue reservation.

## Ethics statement

The studies involving humans were approved by Harbin Medical University Cancer Hospital. The studies were conducted in accordance with the local legislation and institutional requirements. Written informed consent for participation was not required from the participants or the participants’ legal guardians/next of kin in accordance with the national legislation and institutional requirements.

## Author contributions

QL: Conceptualization, Data curation, Investigation, Methodology, Resources, Software, Validation, Writing – original draft, Writing – review & editing. YZ: Methodology, Software, Validation, Writing – review & editing. JZ: Data curation, Methodology, Writing – review & editing. LC: Data curation, Methodology, Writing – review & editing. YY: Supervision, Writing – review & editing. YL: Data curation, Supervision, Writing – review & editing.
